# Medico-Practical Reflexions on the Internal Use of Phosphorus, Particularly in Hemiplegia

**Published:** 1799-06

**Authors:** 


					412 Prof. Br era, on Phofphorus and the Epidemic among Cats.
MATERIA MEDICA.
RifeJJioni medico-praticbe full ufo interna del fosforo, particulamente nelV^'
plegie?Medico -praftic,a I Reflexions on the internal ufe of Phofphorus, Pa,t*'
cularly in hemiplegia.
i t n
by Valeriano-Luigi Brera, 8vo. pp. 4?*
Pwvia.
This diflertation is dedicated to the Society of Medicine at Paris- %
appears that the author preicribed the internal ufe of phofphorus in a cafe ot
hemiphlegia, and that fo far from fucceeding, it was attended with ^e
mod fatal confequences. Indeed he candidly confefles that relying on
obfervations of others, he had the mortification of witneffing the death 0
the perfon to whom it was adminiflered, although he had given it with ^
utmoft precaution; and is certain his want of luccefs could not be attribute
to temerity, inadvertence, or ignorance of the remedy employed.
Profeffor Brera then relates his own obfervations, as well as thofe of
ral other phyfieians, and gives a fliort hiftory of the hemiplegia before iT'er'.
tioned.?The body on difle&ion did not offer the fmallell trace of gangre?e'
but a gas efcaped from the ftomach refembling a white vapour, and frnfH111^
like garlick, which caught fire on the approach of a taper. The coats 0
the ftomach were not in the leaft inflamed; the inteftinal canal was, ho\vev'ej/
a little contra&ed, and fome red fpots were obferved on the inteftines.
appeared that the two firft grains adminiltered by the mouth produced 1 ?
death of the patient; and that thofe adminiftered by giiiters were 11 ^
attended with any ill confequences. The author concludes by expr^1^
his furprife at any phyfician recommending the ufe of fo dreadful a rem^ y
The work, upon the whole, pollefles much information on a ve
interelting fubjett, and well deferves the attention of profeffional men.
f r "
* For further information relative >o the interna! use of phosphorus, we reJC
reader to our third number, p. 783 and foil.

				

